# KBG syndrome

**DOI:** 10.1186/1750-1172-1-50

**Published:** 2006-12-12

**Authors:** Francesco Brancati, Anna Sarkozy, Bruno Dallapiccola

**Affiliations:** 1 CSS Hospital, IRCCS, San Giovanni Rotondo and CSS-Mendel Institute, Rome, Italy; 2 Department of Experimental Medicine and Pathology, University "La Sapienza", Rome, Italy; 3 Department of Biological Sciences and Aging Research Center, Ce.S.I., G. d'Annunzio University Foundation, Chieti, Italy

## Abstract

KBG syndrome is a rare condition characterised by a typical facial dysmorphism, macrodontia of the upper central incisors, skeletal (mainly costovertebral) anomalies and developmental delay. To date, KBG syndrome has been reported in 45 patients. Clinical features observed in more than half of patients that may support the diagnosis are short stature, electroencephalogram (EEG) anomalies (with or without seizures) and abnormal hair implantation. Cutaneous syndactyly, webbed short neck, cryptorchidism, hearing loss, palatal defects, strabismus and congenital heart defects are less common findings. Autosomal dominant transmission has been observed in some families, and it is predominantly the mother, often showing a milder clinical picture, that transmits the disease. The diagnosis is currently based solely on clinical findings as the aetiology is unknown. The final diagnosis is generally achieved after the eruption of upper permanent central incisors at 7–8 years of age when the management of possible congenital anomalies should have been already planned. A full developmental assessment should be done at diagnosis and, if delays are noted, an infant stimulation program should be initiated. Subsequent management and follow-up should include an EEG, complete orthodontic evaluation, skeletal investigation with particular regard to spine curvatures and limb asymmetry, hearing testing and ophthalmologic assessment.

## Disease name and synonyms

KBG syndrome

K.B.G. syndrome

KBG-like syndrome

The name of the syndrome is based on the initials of the first 3 patients reported by Hermann *et al. *in 1975 [1].

## Definition and diagnostic criteria

Currently, the diagnosis of KBG syndrome (OMIM %148050) is uniquely based on clinical findings as no genetic test is available. Despite the lack of defined diagnostic criteria, four cardinal manifestations may be outlined: typical facial dysmorphism, macrodontia of the upper central incisors, skeletal (mainly costovertebral) anomalies and developmental delay. Features observed in more than half of reported patients that may support the diagnosis are short stature, electroencephalogram (EEG) anomalies (with or without seizures) and abnormal hair implantation. Cutaneous syndactyly, webbed short neck, cryptorchidism, hearing loss, palatal defects, strabismus and congenital heart defects are additional findings (Table 1).

**Table 1:** Major and minor clinical features of KBG syndrome.

**CLINICAL CHARACTERISTICS**	**% of KBG patients (counted individuals)**
**Major Features**	
**Facial dysmorphisms**	**100 (45)**
Prominent/high nasal bridge	78
Thin upper lip	71
Anteverted nostrils	62
Brachycephaly/turricephaly	60
Telecanthus/hypertelorism	58
Long philtrum	51
Wide eyebrows	49
Prominent/anteverted ears	42
Mild synophrys	35
Epicanthal folds	26
Ptosis	24
Facial asymmetry	24
**Macrodontia**	100* (42)
with oligodontia	38 (45)
**Skeletal abnormalities**	**100**
Abnormal ribs/vertebrae	83 (35)
Delayed bone age	79 (28)
Short hand tubular bones	78 (40)
Brachy-clinodactylous 5^th ^finger	62 (45)
Abnormal spine curvature	58 (31)
Short femoral necks/hip dysplasia	50 (26)
Sternum abnormalities	29 (44)
Wormian bones in skull	19 (21)
**Cognitive deficits/psychomotor delay**	**91 (45)**
**Short stature (<10°c)**	**77 (45)**
**Abnormal EEG**	**72 (25)**
with seizures	24 (45)
**Abnormal hair implantation**	**58 (45)**
	
**Minor Features**	
**Cutaneous syndactyly, toes II/III**	**46 (45)**
**Webbed/short neck**	**42 (38)**
**Cryptorchidism**	**28 (29)**
**Hearing loss**	**23 (43)**
**Palatal defects (including uvula)**	**18 (38)**
**Strabismus**	**18 (45)**
**Congenital heart defects**	**9 (45)**

*in three patients macrodontia was only reported on interview as premature loss of teeth occurred

## Epidemiology

Since the first description in 1975 of seven affected individuals from three unrelated kindred [1], 26 additional families have been reported so far, leading to a total number of 45 KBG patients [2-15]. These include eight affected individuals with so-called KBG-like syndrome [5,9]. According to Smithson *et al. *[11], patient 2 reported by Zollino and colleagues [15] may be affected by another condition and was not added. It is likely that KBG syndrome is underdiagnosed as many of its features are mild and the diagnosis can be missed in absence of a careful clinical examination.

## Clinical description by organ systems

### Craniofacial

**Figure 1:**
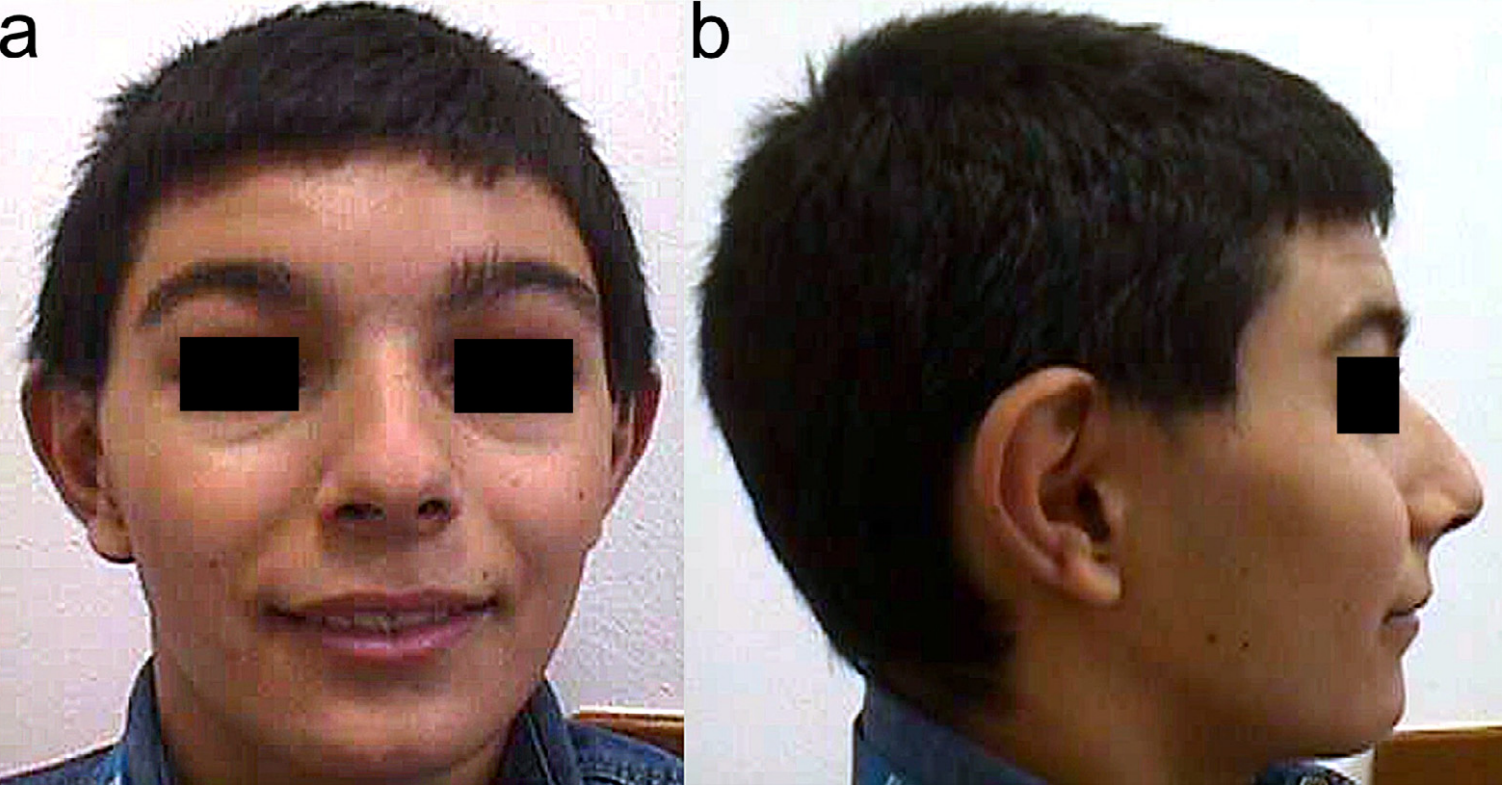
Frontal (a) and lateral (b) view of a KBG patient with typical facial dysmorphisms including low frontal hairline, wide eyebrows with mild synophrys, hypertelorism, prominent and high nasal bridge, anteverted nostrils, long philtrum, thin upper lip and prominent anteverted ears. Macrodontia of upper central incisors is observable.

A characteristic facial appearance has been outlined, which should prompt the clinician to consider this diagnosis (Figure 1a, b). Children have a round face, with wide eyebrows and mild synophrys, hypertelorism, a prominent and high nasal bridge with anteverted nostrils, a long philtrum and a thin upper lip. The cranium may be brachy- turricephalic (microcephaly has been described in some cases), while hairs are often coarse with a low frontal or posterior hairline (a cowlick aspect of the frontal hairline is frequently observed). The ears appear prominent and anteverted. Additional dysmorphic features are telecanthus, epicanthal folds, ptosis and facial asymmetry. The neck is short in nearly half of the patients. The facial appearance may change with age being more triangular in adolescents and adults. However in some patients and, in particular, in affected mothers these findings may be less recognisable.

### Dental

**Figure 2:**
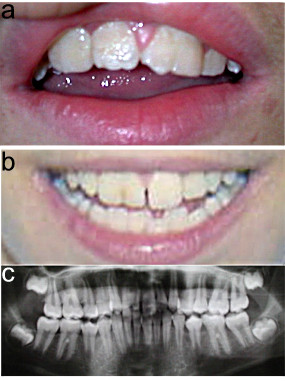
Macrodontia of upper central incisors is a constant feature of KBG syndrome (a). Fusion of upper and lateral right incisors is evident in this other patient on physical (b) and panorex film (c) examination.

The most consistent finding is wide upper central incisors as shown in Figure 2a (defined as a width of >9.2 mm [16]). Macrodontia (or taurodontia) is associated with extra mammelons *i.e. *four or more little notches on the incisal edges of the permanent central maxillary incisors. Although rare, a very peculiar finding is the partial fusion of superior central and lateral incisors, which results in a large cleft tooth (Figure 2b, c). Oligo- or hypodontia may affect 38% of patients ranging from hypoplastic to absent lateral incisors. Premature loss of teeth is frequent in adults, whereas enamel hypoplasia or dental pits occur in about 18% of the cases. An odontogenic cyst of the mandible was reported in a single patient [2].

### Skeletal

**Figure 3:**
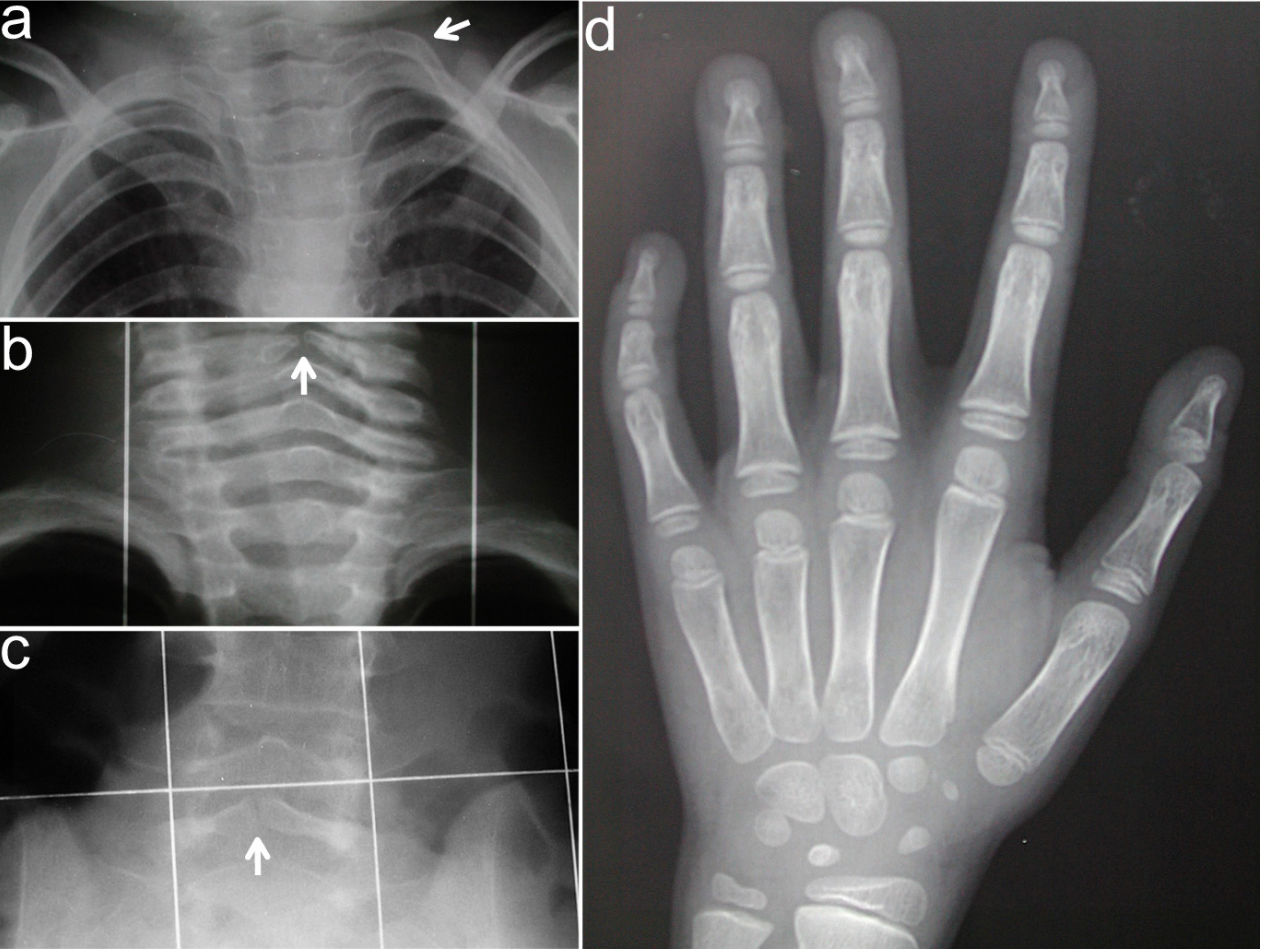
Common skeletal defects observed in KBG syndrome include supernumerary cervical rib (a, arrow), schisis of the posterior arch of cervical (b, arrow) and/or sacral (c, arrow) vertebrae. Left hand X-ray of a 10-year-old male KBG patient (d) showing shortened tubular bones especially of the III-IV-V metacarpus, the I distal and the V middle phalanges with clinodactylous V finger. Bone age is delayed in particular with respect to carpal bones.

Skeletal anomalies occur in all patients and are used as major diagnostic criteria (Figure 3a–d). The most common findings include abnormalities of the vertebrae and ribs. Deficient/absent vertebral arches (schisis or closed spina bifida), block fusions, irregular vertebral end-plates and intervertebral distance, and asymmetry of the medullary canal (involving preferentially the cervical and the lumbosacral tracts of the column), are present in up to 74% of the patients. Cervical ribs are common (46%) and may reflect hyperplasia of the transverse process. Hands are involved with short tubular bones and brachy- or clinodactyly of the 5^th ^fingers, leading to a distinct metacarpo-phalageal profile pattern [2]. Bone age is usually delayed. More than half of the patients display scoliosis or kyphosis and one third have sternum abnormalities mainly *pectus excavatum*. Short femoral necks, hypoplastic iliac bones (sometimes associated with hip dysplasia), and wormian bones of the skull are additional findings. Postaxial hand polydactyly, lower limb asymmetry and short tibia have been observed in single patients [1,2].

### Development and behaviour

All KBG children have been considered as delayed to some extent. Psychomotor development is delayed with respect to motor milestones with age at sitting with support ranging from 6 to 16 months (median 9 months) and age at walking from 14 to 60 months (median 20.5 months). Language skills are markedly retarded with first words at 47 ± 34 months (median 36 months). Three patients achieved the ability to speak only at age 5–11 years [1,6,11]. Deafness, which may present with delay in language, should be ruled out. In general, mental retardation ranges from mild to severe, and assessed intelligence quotient (IQ) ranges between 40 and 74. However, overall this is not a constant feature. In particular, affected mothers show an IQ at the lower limit of the normal range with only poor communication skills and intersocial relationships. The great majority of patients are in the mild to moderate range for mental handicap needing special educational assistance but preserving self-help skills. Behavioural disturbances are common, in particular hyperactivity (five patients have been treated with methylphenidate) but also attention deficit and easy frustration.

### Neurological

Non-specific EEG anomalies occur in 72% of the patients and one quarter of them develop seizures (transient childhood epilepsy, grand mal seizures). Central nervous system (CNS) structural abnormalities are rare and include hypoplastic cerebellar vermis, enlarged cysterna magna, Chiari malformation type I, and meningomyelocele, which have been reported in single patients [2,6,15].

### Growth

Birth weight is usually normal while length is often below the 3^rd ^centile. Seventy-seven percent of KBG patients display proportionate short stature (<10^th ^centile), with respect to the height of the parents and sibs. However, arm span exceeding stature was noted in several patients. The adult final height cannot be established with certainty, based on the small number of reported individuals. The mean stature of eight available adults with short stature was 149 cm for females and 153.6 cm for males, while two adult patients displayed normal height. Short stature represents a major and primary manifestation of this condition. Studies of growth hormone (GH) and insulin growth factor-1 (IGF-1) levels gave normal results [[13], personal observation].

### Otolaryngologic and hearing

Mild to moderate hearing loss is documented in 23% of the patients. When reported, deafness can be either conductive (four patients), mixed (three patients) or sensorineural (one patient). Of note, recurrent otitis media was frequently recorded and triggered deafness in several patients.

Eighteen percent of the patients display palatal and pharyngeal defects, including soft cleft palate, bifid uvula, notching of the posterior border of the hard palate, and velopharyngeal insufficiency sometimes requiring surgical correction. Hypertrophic tonsils and adenoid tissues, causing recurrent upper airway infections/otitis and requiring adenoid-tonsillectomy, are frequent findings. Some subjects have a dysphonic voice (either hypernasality or low-pitched voice).

### Other

Around 28% of patients have undescended testes. Cryptorchidism may partly explain the reduced fitness in males and the preferential transmission of the syndrome by affected mothers, as seen in other conditions such as Noonan syndrome.

Ocular defects are found more frequently than in the general population. Strabismus occurs in 18% of the cases, while a broad range of abnormalities including, congenital bilateral cataract, high grade myopia/aspecific visual impairment and megalocornea have been reported in single patients [2,8,15].

Congenital heart defects (CHD) and abnormalities of the great arteries are not common (9%) and include stenosis of the left pulmonary artery, ventricular septal defect, bicuspid aortic valve, partial atrioventricular canal defect [2,3,10].

## Inheritance and genetic counselling

The ten known families with KBG syndrome display vertical transmission of the disease with marked variability of expression. It is predominantly mothers (who often show a milder clinical picture), and not fathers who transmit the disease (4:1 ratio). Overall, males are affected almost twice as often as females (29 to 16) and among the 19 sporadic patients, only 5 are females. These observations prompted some authors to suggest X-linked inheritance in some cases. However, male-to-male transmission has been reported, weakening this hypothesis [1,13]. A careful clinical examination of the parents (especially the mothers) is hence mandatory, and if one parent is affected a one in two recurrence risk should be advised.

## Management

KBG syndrome is usually not associated with severe medical complications. The diagnosis is rarely achieved before the upper permanent central incisors have erupted at age 7–8 years. Accordingly, a number of features of the syndrome are assessed by routine paediatric evaluation. The clinical geneticist should ensure the proper management of congenital anomalies, in particular cryptorchidism, palatal defects, CHD and CNS malformations.

At time of diagnosis, a full neuropsychological assessment should be done in particular if delays are diagnosed. It is important to promptly initiate infant stimulation, early intervention, and special education programs, as well as early speech therapy. Severe behaviour problems would benefit from specific psychopharmacological medications.

EEG may need to be carried out and anticonvulsant therapy is indicated for a seizure disorder.

Referral to an orthodontist is recommended once permanent teeth erupt, to assess malocclusion and intervention. Regular dental visits are encouraged, also for the treatment of early dental decay.

A full skeletal investigation (including bone age in paediatric patients) should be carried out in all patients with a suspected or proven diagnosis. Among the skeletal defects, only pathologic spine curvatures and eventual limb length discrepancies require specialist evaluation and treatment. In general, other vertebral anomalies are asymptomatic and do not require treatment.

It is useful to evaluate growth velocity using standard charts and if deficiency is noted, common causes of short stature should be promptly ruled out. No specific therapy is available for the treatment of short stature of unknown cause.

Particular care should be addressed to otitis media which could result in hearing loss. Accordingly, aggressive antibiotic therapy is indicated while transtimpanic drainage is recommended in recurrent forms. Comprehensive audiological evaluations are suggested in all newly diagnosed patients to determine the nature and extent of the possible hearing loss. Annual testing is useful to monitor the development and progression of deafness. If necessary, hearing aids should be offered.

Finally, an initial detailed visual assessment is required and periodic ophthalmologic evaluations should be performed if anomalies are found.

## Abbreviations

CHD: congenital heart defect;

CNS: central nervous system;

GH: growth hormone;

EEG: electroencephalogram;

IQ: intelligence quotient;

IGF-1: insulin growth factor-1.

## Competing interests

The author(s) declare that they have no competing interests.

## Authors' contributions

FB and BD both contributed to the conception of the study, participated in its design and coordination and drafted the manuscript. AS participated in the design of the study and performed the statistical analysis. All authors read and approved the final manuscript.

## References

[B1] Herrmann J, Pallister PD, Tiddy W, Opitz JM (1975). The KBG syndrome-a syndrome of short stature, characteristic facies, mental retardation, macrodontia and skeletal anomalies. Birth Defects Orig Artic Ser.

[B2] Brancati F, D'Avanzo MG, Digilio MC, Sarkozy A, Biondi M, De Brasi D, Mingarelli R, Dallapiccola B (2004). KBG syndrome in a cohort of Italian patients. Am J Med Genet A.

[B3] Devriendt K, Holvoet M, Fryns JP (1998). Further delineation of the KBG syndrome. Genet Couns.

[B4] Dowling PA, Fleming P, Gorlin RJ, King M, Nevin NC, McEntagart M (2001). The KBG syndrome, characteristic dental findings: a case report. Int J Paediatr Dent.

[B5] Fryns JP, Haspeslagh M (1984). Mental retardation, short stature, minor skeletal anomalies, craniofacial dysmorphism and macrodontia in two sisters and their mother. Another variant example of the KBG syndrome?. Clin Genet.

[B6] Maegawa GH, Leite JC, Felix TM, da Silveira HL, da Silveira HE (2004). Clinical variability in KBG syndrome: report of three unrelated families. Am J Med Genet A.

[B7] Mathieu M, Helou M, Morin G, Dolhem P, Devauchelle B, Piussan C (2000). The KBG syndrome: an additional sporadic case. Genet Couns.

[B8] Novembri A, Franchini F, Calzolari C, Vieri PL, Giovannucci ML (1983). K.G.B. syndrome: review of the literature and presentation of a case. Arch Putti Chir Organi Mov.

[B9] Parloir C, Fryns JP, Deroover J, Lebas E, Goffaux P, van den Berghe H (1977). Short stature, craniofacial dysmorphism and dento-skeletal abnormalities in a large kindred. A variant of K.B.G. syndrome or a new mental retardation syndrome. Clin Genet.

[B10] Rivera-Vega MR, Leyva Juarez N, Cuevas-Covarrubias SA, Kofman-Alfaro SH (1996). Congenital heart defect and conductive hypoacusia in a patient with the KBG syndrome. Clin Genet.

[B11] Smithson SF, Thompson EM, McKinnon AG, Smith IS, Winter RM (2000). The KBG syndrome. Clin Dysmorphol.

[B12] Soekarman D, Volcke P, Fryns JP (1994). The KBG syndrome: follow-up data on three affected brothers. Clin Genet.

[B13] Tekin M, Kavaz A, Berberoglu M, Fitoz S, Ekim M, Ocal G, Akar N (2004). The KBG syndrome: confirmation of autosomal dominant inheritance and further delineation of the phenotype. Am J Med Genet A.

[B14] Tollaro I, Bassarelli V, Calzolari C, Franchini F, Giovannucci Uzielli ML, Vieri PL (1984). Dento-maxillo-facial anomalies in the KBG syndrome. Minerva Stomatol.

[B15] Zollino M, Battaglia A, D'Avanzo MG, Della Bruna MM, Marini R, Scarano G, Cappa M, Neri G (1994). Six additional cases of the KBG syndrome: clinical reports and outline of the diagnostic criteria. Am J Med Genet.

[B16] Moyers RE (1958). Handbook of Orthodontics.

